# Ultrarare Missense Variants Implicated in Utah Pedigrees Multiply Affected With Schizophrenia

**DOI:** 10.1016/j.bpsgos.2023.02.002

**Published:** 2023-02-16

**Authors:** Cathal Ormond, Niamh M. Ryan, Elizabeth A. Heron, Michael Gill, William Byerley, Aiden Corvin

**Affiliations:** a Neuropsychiatric Genetics Research Group, Department of Psychiatry, Trinity College Dublin, Dublin, Ireland; b Department of Psychiatry and Behavioral Sciences, University of California, San Francisco, California

**Keywords:** ATP2B2, Cosegregation, Pedigree, SCHEMA, Schizophrenia, Whole genome sequencing (WGS)

## Abstract

**Background:**

Recent work from the Schizophrenia Exome Sequencing Meta-analysis (SCHEMA) consortium showed significant enrichment of ultrarare variants in schizophrenia cases. Family-based studies offer a unique opportunity to evaluate rare variants because risk in multiplex pedigrees is more likely to be influenced by the same collection of variants than an unrelated cohort.

**Methods:**

Here, we examine whole genome sequencing data from 35 individuals across 6 pedigrees multiply affected by schizophrenia. We applied a rigorous filtering pipeline to search for classes of protein-coding variants that cosegregated with disease status, and we examined these for evidence of enrichment in the SCHEMA dataset. Additionally, we applied a family-based consensus approach to call copy number variants and screen against a list of schizophrenia-associated risk variants.

**Results:**

We identified deleterious missense variants in 3 genes (*ATP2B2*, *SLC25A28*, and *GSK3A*) that cosegregated with disease in 3 of the pedigrees. In the SCHEMA, the gene *ATP2B2* shows highly suggestive evidence for deleterious missense variants in schizophrenia cases (*p* = .000072). *ATP2B2* is involved in intracellular calcium homeostasis, expressed in multiple brain tissue types, and predicted to be intolerant to loss-of-function and missense variants.

**Conclusions:**

We have identified genes that are likely to increase schizophrenia risk in 3 of the 6 pedigrees examined, the strongest evidence being for a gene involved in calcium homeostasis. Further work is required to examine other classes of variants that may be contributing to disease burden.

Schizophrenia is a clinically heterogeneous brain disorder that affects how people perceive and understand the world around them. Existing treatments are partially effective in most people, but outcomes are often poor, and patients die 12–15 years before the average population (1). The underlying etiology is poorly understood, but most of the variation in risk between individuals is genetically mediated, involving a spectrum of risk alleles from many common alleles of small effect to rare copy number and coding variants of larger effect. To date, more than 270 common variants have been confirmed by the Psychiatric Genomics Consortium (PGC), but collectively these explain only a small fraction of risk and likely represent <10% of all common contributory variants (2). Twelve known copy number variants (CNVs) substantially increase individual risk by 2- to 60-fold (3) but are only present in about 2% of patients (4). The causal mechanism underlying most of these risk loci is still to be determined as the associated loci often overlap multiple genes or regulatory features. Identifying rare coding variants that contribute to risk may be particularly important in understanding the complex molecular network involved and in developing better treatments for schizophrenia.

Recently, the Schizophrenia Exome Sequencing Meta-analysis (SCHEMA) consortium reported 10 genes in which the burden of ultrarare variants (URVs) was significantly higher in schizophrenia cases (5). Their study also showed that schizophrenia cases carry an excess of pathogenic URVs in loss-of-function (LoF) intolerant genes compared with controls, even when these 10 genes were removed. Highly deleterious missense variants had as strong a signal as protein-truncating variants. These results suggest that many more genes in which URVs contribute to schizophrenia risk are yet to be discovered. Family-based studies offer a unique opportunity to identify and evaluate rare variants conferring risk for schizophrenia because densely affected pedigrees are more likely to be influenced by the same subset of variants compared with an unrelated cohort. Ultrarare, family-private variants have been shown to be enriched in multiplex compared with simplex pedigrees in autism, indicating that they are good candidates for novel gene discovery (6). Historically, linkage analysis has been used to identify candidate causal genes or regions in pedigrees, but this usually requires data from many individuals across several generations. Instead, a cosegregation analysis can be substituted to analyze variants present in appropriate individuals within each family. Here, we examine whole genome sequencing (WGS) data from 6 multiplex pedigrees affected by schizophrenia to identify URVs likely contributing to the phenotypic risk.

## Methods and Materials

### Sample Procurement and Assessment

Methods used in sample ascertainment and assessment were approved by the University of Utah Institutional Review Board. Multiplex pedigrees were identified by screening hospitalized patients with diagnoses of schizophrenia. Following written informed consent, subjects were interviewed by a clinician using the Schedule for Affective Disorders and Schizophrenia-Lifetime Version (7). Medical records were obtained for any individual who received psychiatric care. The interview results and any medical records were then presented to a diagnostic panel comprising 2 clinicians who played no role in ascertainment or assessment. Consensual diagnoses were made using Research Diagnostic Criteria (8). Six pedigrees of predicted European ancestry in which schizophrenia was the dominant phenotype were selected from the cohort (Figure S1).

### Whole Genome Sequencing

#### Batch 1

Twenty-six samples across the 6 pedigrees had been selected previously for WGS. DNA concentrations were quantified by Qubit (Thermo Fisher Scientific), and the quality of DNA was determined by agarose gel electrophoresis. WGS was performed by MedGenome, Inc. on an Illumina HiSeqX to an average depth of coverage of at least 30× per sample. Some of these samples had been aligned to the GRCh38 reference genome using the Sentieon Genomics proprietary pipeline but were realigned locally to match additional data from these pedigrees.

#### Batch 2

Twelve additional samples were selected for WGS. DNA concentrations were quantified using NanoDrop (Thermo Fisher Scientific), and the quality of DNA was determined by agarose gel electrophoresis. WGS was performed by Edinburgh Genomics (Clinical Genomics) on an Illumina HiSeqX to an average depth of coverage of at least 30× per sample.

### Data Preprocessing and Quality Control

All binary alignment map (BAM) files from batch 1 were converted to paired-end FASTQ files using the Picard toolkit (9) so they could be realigned using the same pipeline and to the same reference genome as batch 2. This involved reverting the BAM to remove all mapping information, fixing errors in mate pairs, removing reads without matching pairs, and randomizing the reads to remove any potential downstream bias resulting from the original sort order. All FASTQ files were examined using FastQC and samtools (10) to identify DNA contamination or degradation. Reads were aligned to the GRCh38 reference genome (GCA_000001405.15, including decoy, human leukocyte antigen, and alternative contigs) using BWA-MEM (Burrows-Wheeler aligner, maximal exact matches) (11), following the GATK version 3 Best Practices (12). This involved marking polymerase chain reaction duplicates with Picard, base quality score recalibration, local realignment of reads around indels, and variant calling with HaplotypeCaller (GATK version 3.8-0-ge9d806836). All samples were jointly genotyped using the GenotypeGVCFs module from GATK using default parameters.

After calling genotypes, variants whose depth of coverage was 5 standard deviations greater than the average depth of coverage across all sites were removed. Variants were then split by type (single nucleotide variants [SNVs], indels, and others), retaining those on the standard 23 pairs of chromosomes. Variant quality score recalibration was applied to SNVs and indels separately, using truth-sets provided in the GATK resource bundle. A tranche sensitivity threshold of 99.9% for SNVs and 99.0% for indels was used to remove low-confidence variant sites. For variants other than SNVs and indels, the following hard filters were applied: QualByDepth < 2.0; ReadPosRankSum < −20.0; FisherStrand > 200.0; StrandOddsRatio > 10.0. Multiallelic sites were split into multiple biallelic sites with bcftools norm (13). If any sample had genotype quality < 20.0 or read depth < 10.0, the genotype for that sample was set to missing (14). Finally, variants marked with a filter and variants with missingness > 20% were removed.

The software Peddy (15) was applied to all samples jointly to perform the following pedigree and data checks: 1) relatedness discordance; 2) sex discordance; 3) low median coverage; and 4) ancestry clustering by a principal component analysis based on 1000 Genomes Project data (16). To investigate the presence of batch effects and technological stratification, the software XPAT (17) was applied to all samples. Given the minimum sample requirements for XPAT, additional families recruited from the same cohort and sequenced in the same batches were included.

### SNV and Indel Prioritization

SnpSift version 5.0 (18) was used to identify private variants in families to reduce the computation burden of annotation. Here, private indicates that the variant is present in one family and absent from all other families. We considered all individuals with a diagnosis of schizophrenia to be cases and individuals with no diagnosis as controls. Next, variants were retained if there were no Mendelian violations and they followed either a full cosegregation pattern (carried by all in-family cases, absent from all in-family controls, and absent from all marry-in samples) or a reduced cosegregation pattern (carried by all but one in-family cases, absent from all in-family controls, and absent from all marry-in samples). Custom JavaScript code was added to the FilterVcf module from Picard to identify the case/control status and in-family/marry-in status of samples for the cosegregation filter. We removed variants not present in the coding sequence of a protein-coding gene, as defined by the RefSeq ncbiRefSeqCurated table (19), downloaded from the University of California, Santa Cruz Table Browser (20).

The Variant Effects Predictor version 97.0 (21) was used to annotate each variant, taking the canonical transcript of that gene. Where variants overlapped multiple genes, we examined the canonical transcript of each gene separately. As part of the annotation, we included the Genome Aggregation Database (gnomAD) version 2.1.1 exome allele frequencies (22) and the database of nonsynonymous functional prediction (dbNSFP) version 4.1 (23) from which several deleteriousness metrics were extracted, namely MPC (missense badness, PolyPhen2, and constraint) (24), SIFT version 2.2 (25), PolyPhen2 version 2.2.2 (26), and CADD version 1.6 (27). Gene-based probability of LoF intolerance scores (28), missense *Z* scores (29), and LoF-observed/expected upper bound fraction scores (22) calculated from gnomAD allele frequencies were also annotated from dbNSFP. Custom bash code was used to extract the variant-level scores from dbNSFP that corresponded to the appropriate transcript where scores for different transcripts were supplied.

To prioritize variants likely to be implicated in schizophrenia based on the SCHEMA work, we retained those that satisfied the following: 1) ultrarare in gnomAD (minor allele count ≤ 5 across all samples); 2) either protein-truncating variants (i.e., frameshift, stop gain, or splice acceptor/donor); or predicted-deleterious missense variants (MPC > 2); and 3) present in a highly LoF-intolerant gene (probability of LoF intolerance > 0.9).

### CNV Calling

We used a family-based consensus of 4 software tools to call CNVs from the BAM files (Figure S5). CNVs were called by tools derived from 2 classes of calling methods: CNVnator (30) and ERDS (31) (read depth–based callers); LUMPY (32) and Manta (33) (paired-end/split-read based callers). A collapsing strategy was applied to raw CNV calls to eliminate multiple calls which represent the same site, similar to that described in Trost *et al.* (34). Then, sites that overlapped reciprocally by 50% were merged, first considering within-method call sets (e.g., LUMPY vs. Manta) and then between the resulting across-method call sets. Finally, sites for which over 50% of their length comprised repeat and low complexity regions were removed. Repeat and low complexity regions are defined in Trost *et al.* (34) as 1) assembly gaps (University of California, Santa Cruz gap table); 2) segmental duplications (University of California, Santa Cruz genomicSuperDups table); and 3) the pseudoautosomal regions of the sex chromosomes.

Within each pedigree, we removed CNV calls that were identified by one calling method and were only found in one individual. The resulting calls have the support of either at least two calling methods or multiple individuals in the same pedigree. For variants identified by one tool and present in multiple related samples, if the proportion of reads supporting an event at the breakpoints was low, such calls were removed.

## Results

### WGS Data

Thirty-eight samples across 6 pedigrees were sent for sequencing at a minimum average depth of coverage of 30× (Methods and Materials) (Figure S1). Two samples (K1501_9 and K1527_33) failed sequencing due to low-quality DNA. Additionally, sample K1524_3 was found to be unrelated to their 4 siblings and was removed. The remaining 35 samples passed all pedigree-related checks and were carried forward for analysis (Table 1). A principal component analysis confirmed that all samples clustered with European individuals from the 1000 Genomes Project (Figure S2). XPAT did not reveal any evidence of technological stratification or batch effects across the samples, with only family-based clustering observed (Figures S3 and S4).

**Table 1:** The Total Number of Individuals Sequenced Who Were Retained for Analysis, Broken Down by Diagnosis and Whether They Were Members of the Family (In-Family) or Marry-In Samples

Pedigree	Total	In-Family		Marry-In
		SCZ	UN	
K1480	5	4	––	1
K1494	5	4	––	1
K1501	7	4	2	1
K1524	4	4	––	––
K1527	6	5	––	1
K1546	8	5	2	1
Total	35	26	4	5

SCZ, schizophrenia; UN, unaffected.

### Prioritized SNVs

**Figure 1:**
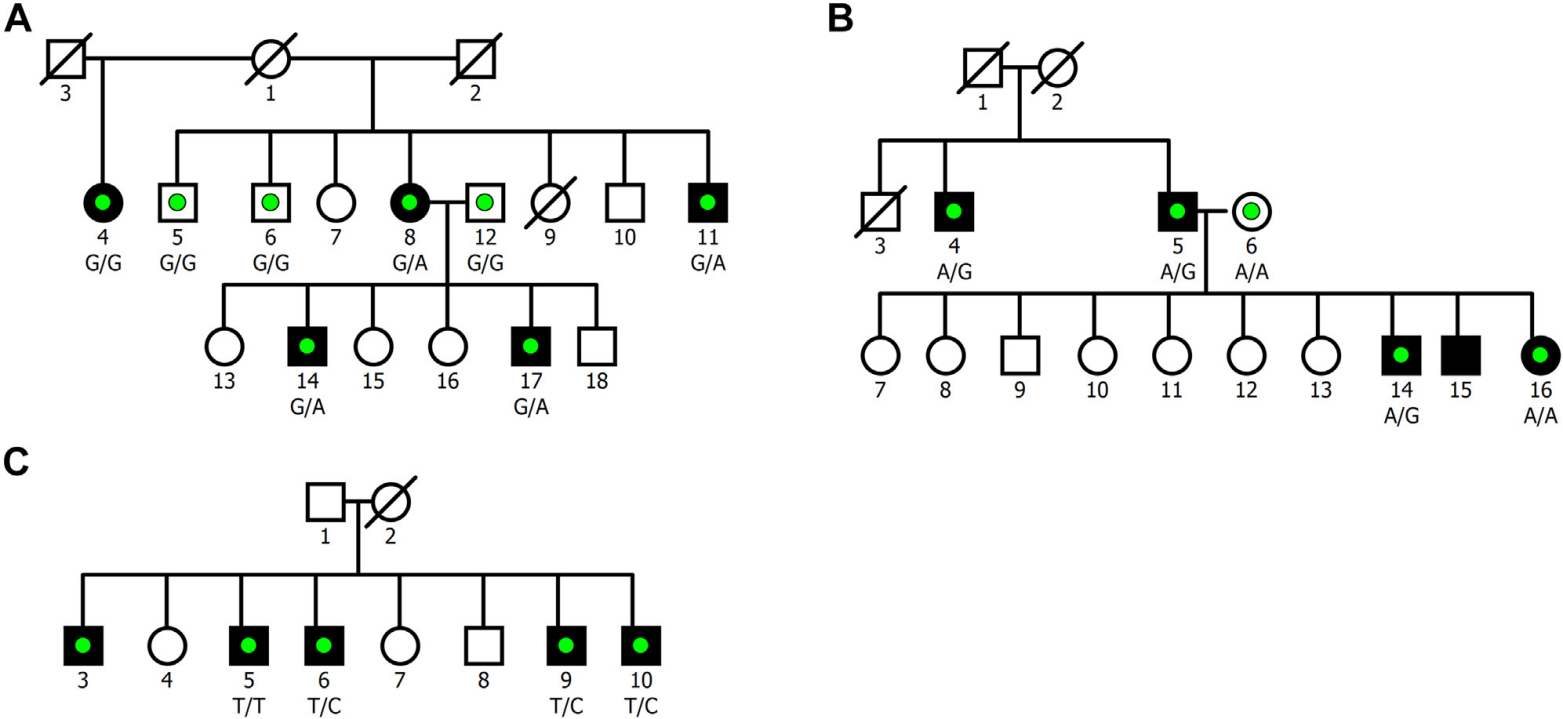
Pedigree images of the families that harbor an ultrarare missense variant with reduced cosegregation. Fully shaded boxes denote individuals with a diagnosis of schizophrenia, and sequenced individuals are marked with a colored dot. The genotype of the identified single nucleotide variant is shown beneath all sequenced individuals that were carried forward for analysis. Additional details for the variants are given in Table 3. **(A)** K1546 (*ATP2B2*:p.Arg588Cys); **(B)** K1494 (*GSK3A*:p.Ile377Thr); and **(C)** K1524 (*SLC25A28*:p.Ile341Val).

In total 11,509,434 variants passed all quality control measures following the joint genotyping of all samples, of which 3,069,960 were private to one of the families. After applying the main prioritization filters, no ultrarare, functionally relevant variants were identified that had full cosegregation with cases (Table 2). However, 3 deleterious missense URVs (class II variants from SCHEMA: 2 ≤ MPC < 3) that followed a reduced cosegregation pattern were identified, one in each of pedigrees K1546, K1494, and K1524 (Table 3; Figure 1).

**Table 2:** The Number of Variants Remaining After Each Stage of the Prioritization Process

Description	No. of Variants	
Quality Control Filters	11,509,434	
Family-Private Variants	3,069,960	

	Full	Reduced
Cosegregation Pattern	57,253	172,748
In the Coding Sequence	515	1904
Ultrarare in gnomAD	50	210
Functional Relevance	0	10
LoF-Intolerant Gene	0	3

Counts on the left are for full cosegregation and on the right are for reduced cosegregation.

gnomAD, Genome Aggregation Database; LoF, loss-of-function.

**Table 3:** Details of the 3 Prioritized Variants With Reduced Cosegregation

Pedigree	Chr	Position	Variant	Gene	Exon	HGVSp	MAC	MPC	CADD	SIFT	PolyPhen2
K1546	3	1,036,0021	G > A	*ATP2B2*	13/23	R588C	1	2.23	31.0	Damaging	Deleterious
K1524	10	99,610,923	T > C	*SLC25A28*	4/4	I341V	0	2.11	25.6	Damaging	Deleterious
K1494	19	42,232,651	A > G	*GSK3A*	9/11	I377T	0	2.39	26.9	Damaging	Deleterious

Positions are given on GRCh38. Included are the protein sequence ID (HGVSp), the MAC from gnomAD (exome), and several deleteriousness prediction metrics.

Chr, chromosome; MAC, minor allele count; MPC, missense badness, PolhPhen2, and constraint.

### Schizophrenia-Associated CNVs

We called CNVs in all samples using a consensus of 4 calling tools to rule out any variants with a known association with schizophrenia (Table S1). Several such variants were initially identified in the cohort, most were excluded when examining the level of support at the breakpoints (Table S2). Only 1 rare variant was retained, a duplication on chromosome 16p11.2 in sample K1524_5. This CNV was called by both read depth–based callers and was not observed in any other samples in this pedigree.

## Discussion

We examined WGS data from 35 individuals across 6 pedigrees recruited from Utah that were multiply affected by schizophrenia. In the absence of sufficient number of individuals to perform linkage analysis, we performed a cosegregation analysis to identify variants that are likely to increase disease burden. Following recent work from the SCHEMA consortium, we investigated the presence of ultrarare, deleterious variants in LoF-intolerant genes. While no fully cosegregating pathogenic URVs were found, we did observe 3 missense variants with a reduced cosegregation pattern in 3 families. All 3 variants were predicted to be deleterious by additional pathogenicity metrics (Table 3). None of the 3 genes survived false discovery rate correction in the reported SCHEMA analysis, but there was a suggestive excess of the same class of missense variants at *ATP2B2* in the schizophrenia cases compared with controls in the SCHEMA dataset (Table 4). Additionally, the variant in *ATP2B2* identified here was observed only in 1 schizophrenia sample in the SCHEMA dataset. *ATP2B2* has the highest missense *Z* score of the 3 genes, indicating that it is the most intolerant to missense variants.

**Table 4:** Gene-Level Constraint Information From gnomAD and Results for Class II Variants From the SCHEMA Analysis

Gene	Constraint			SCHEMA (Class II)	
	pLI	Missense *Z* Score	LOEUF	OR	*p* Value
*ATP2B2*	1.00	4.55	0.15	1.920	.000719
*SLC25A28*	0.93	2.92	0.37	0.617	.744000
*GSK3A*	1.00	3.22	0.13	0.830	.835000

Information from gnomAD includes the pLI Score (28), the missense *Z* score (29), and the LOEUF metric (22). Results from the SCHEMA analysis (5) include odds ratio and *p* value.

gnomAD, Genome Aggregation Database; LOEUF, loss-of-function observed/expected upper bound fraction; OR, odds ratio; pLI, probability of loss-of-function intolerance; SCHEMA, Schizophrenia Exome Sequencing Meta-analysis.

*ATP2B2* is a member of the plasma membrane Ca^2+^ ATPase (PMCA) protein family, which is involved in intracellular calcium homeostasis (19). It is found to be expressed in multiple brain tissue types in the GTEx project (35). In a genome-wide association meta-analysis of autism spectrum disorder and schizophrenia, an intronic variant in this gene (rs9879311) was found to be genome-wide significant (36). Additionally, damaging de novo variants in *ATP2B2* have been shown to be significantly enriched in autism spectrum disorder cases compared with unaffected siblings in a Japanese cohort (37). A protein-protein interaction analysis of genes implicated in schizophrenia from both rare variants (CNVs and de novo SNVs) and common single nucleotide polymorphisms pointed to NMDA receptor genes as having significant combined effects between rare and common variants (38). *ATP2B2* was found to be connected to the core members of this NMDA receptor interactome. A paralog of this gene is *ATP2A2*, which is a member of the sarco/endoplasmic reticulum CA2+ ATPase (SERCA) protein family. Variants in this gene cause Darier disease, which is known to increase risk for schizophrenia and bipolar disorder (39). Fine-mapping of the significant loci from the PGC schizophrenia phase 3 genome-wide association study identified an intronic variant of *ATP2A2* as highly probable of being causal (2).

*SLC25A28* is part of the mitochondrial carrier subgroup of the SLC gene family. It is a mitochondrial iron transporter that mediates iron uptake and is expressed in most tissue types, including several brain tissues (35). There is no previous evidence of association between *SLC25A28* and schizophrenia or related disorders. *GSK3A* is one of the two isoforms of the GSK-3 protein kinase and is expressed in multiple brain tissues (35). Lithium, used to treat bipolar disorder, inhibits the activity of the paralog of this gene (*GSK3B*) (40). The variant in this gene was also present in the SCHEMA analysis, where one allele was observed in an individual with schizophrenia. Only 1 individual across the 6 families was found to carry a rare, schizophrenia-associated CNV: a duplication on chromosome 16p11.2 was called in sample K1524_5. This individual was the only sample in the pedigree not to carry the URV in *SLC25A28*.

This study represents an exploratory analysis of these pedigrees to screen for classes of gene-affecting variants that are known to be associated with schizophrenia. Full cosegregation would provide the strongest evidence for candidate causal variants, but within each pedigree, the inheritance patterns of the 3 SNVs are consistent with a variant inherited from a common ancestor of most of the affected individuals. The majority of the unaffected individuals from these 3 pedigrees were last contacted after the typical age of onset of schizophrenia, so are likely to have remained as such (Table S3). It is worth noting that the number of samples sequenced in each pedigree is modest, so additional evidence could be gained by the availability of other affected and unaffected family members. Further work is needed to examine other fully cosegregating variants, as well as variants outside the protein-coding regions. We employed strict cut-off thresholds for our filtering and acknowledge that other analytical strategies may identify plausible candidate causal variants missed by the filtering approach.

The methodology implemented here is robust and follows practices established by large-scale genomics consortia, but the SNVs and CNV identified need to be validated with additional sequencing methods. Given how rare these variants are in the population, large sample sizes are required in the discovery analysis to achieve statistical significance, and future work by the SCHEMA consortium may implicate *ATP2B2* as a schizophrenia risk gene.

## Supplementary Material

Supplementary Material

Key Resources Table
