# Supplementary material for: Ultrarare Missense Variants Implicated in Utah Pedigrees Multiply Affected With Schizophrenia
Source: Biol Psychiatry Glob Open Sci. 2023 Feb 16;3(4):797–802. doi: 10.1016/j.bpsgos.2023.02.002 (PMC10593875; doi:10.1016/j.bpsgos.2023.02.002)
Supplement: Supplementary Material [file mmc1.pdf]

SUPPLEMENTARY INFORMATION

Ultra-Rare Missense Variants Implicated in Utah Pedigrees Multiply Affected With Schizophrenia

Ormond *et al.*

Supplementary Tables

Supplementary Table 1: A list of 12 population-rare CNVs, with a significant association with schizophrenia, taken from (1). DEL: deletion; DUP: duplication.

Chr	Start	End	Locus	Type	OR (95% CI)	Notes
chr1	147068456	147917873	1q21.1	DUP	3.45 (1.92 - 6.20)	
				DEL	8.35 (4.65 - 14.99)	
chr2	49922862	51032862	2p16.3	DEL	9.01 (4.44 - 18.29)	*NRXN1* gene
chr3	196003129	197613129	3q29	DEL	57.65 (7.58 - 438.44)	
chr7	73325996	74725666	7q11.23	DUP	11.35 (2.58 - 49.93)	
chr15	22783067	23073067	15q11.2	DEL	2.15 (1.71 - 2.68)	
chr15	24574853	28184854	15q11q13	DUP	13.20 (3.72 - 46.77)	Prader-Willi/Angelman syndrome
chr15	30837797	32187799	15q13.3	DEL	7.52 (3.98 - 14.19)	
chr16	21938679	22418679	16p12.1	DEL	3.3 (1.61 - 7.05)	
chr16	28808679	29038679	16p11.2, distal	DEL	20.6 (2.6 - 162.2)	
chr16	29628679	30188679	16p11.2, proximal	DUP	11.52 (6.86 - 19.34)	
chr22	19032487	20272477	22q11.2	DEL	INF (28.27 - INF)	

Supplementary Table 2: CNVs with known psychiatric association identified in the cohort with a breakdown of which of the four callers identified the CNV (C – CNVnator; E – ERDS; L – LUMPY; M – Manta), and for the CNVs identified by LUMPY, the number of reads that support the event (SU), the read depth at the start of the CNV (DP_S) and the read depth at the end of the CNV (DP_E). The start and end points are given for the GRCh38 reference genome. CNVs called by a consensus of callers were retained, and CNVs called by one caller with low level of support were rejected.

Pedigree	Sample	Chr	Start	End	Type	Locus	Caller				LUMPY Support			Decision
							C	E	L	M	SU	DP_S	DP_E	
K1501	K1501_12	chr15	22775323	28847756	DUP	15q11			×		4	44	56	Reject
	K1501_15	chr15	22657718	28730831	DUP	15q11			×		4	40	31	Reject
K1524	K1524_5	chr15	30631954	32621480	DEL	15q13.3			×		4	28	56	Reject
	K1524_9	chr15	30631898	32621467	DEL	15q13.3			×		4	22	23	Reject
	K1524_5	chr16	29774001	30223000	DUP	16p11.2	×	×			-	-	-	Keep
K1546	K1546_4	chr3	195945211	197641359	DEL	3q29			×		4	68	106	Reject
	K1546_17	chr3	195945145	197641370	DEL	3q29			×		4	134	107	Reject
	K1546_8	chr3	195945163	197641349	DEL	3q29			×		4	104	66	Reject
	K1546_11	chr3	195945211	197641365	DEL	3q29			×		7	91	112	Reject

Supplementary Table 3: For the three pedigrees carrying a prioritized variant, the age of onset for schizophrenia cases, and the age of last contact.

Family ID	Individual ID	Age of Onset	Age Last Contact
K1494	3	N/A	74
	4	33	73
	5	19	70
	6	N/A	66
	7	N/A	47
	8	N/A	46
	9	N/A	45
	10	N/A	44
	11	N/A	43
	12	N/A	41
	13	N/A	40
	14	28	39
	15	22	37
	16	17	36
K1524	1	N/A	72
	2	N/A	63
	3	21	43
	4	N/A	40
	5	20	38
	6	20	38
	7	N/A	36

	8	N/A	35
	9	17	27
	10	21	26
1546	4	32	68
	5	N/A	66
	6	N/A	65
	7	N/A	61
	8	18	58
	12	N/A	58
	13	N/A	35
	14	18	34
	15	N/A	33
	16	N/A	31
	17	17	29
	18	N/A	27
	10	N/A	55
	11	19	52

Supplementary Figures

Supplementary Figure 1: Pedigree diagrams for the six pedigrees selected for analysis. Individuals fully shaded have a diagnosis of schizophrenia, and individuals with the top left quarter shaded have a diagnosis of OCD. Individuals marked with a coloured dot (red: Batch 1; blue: Batch 2; gray: failed WGS) were selected for WGS.

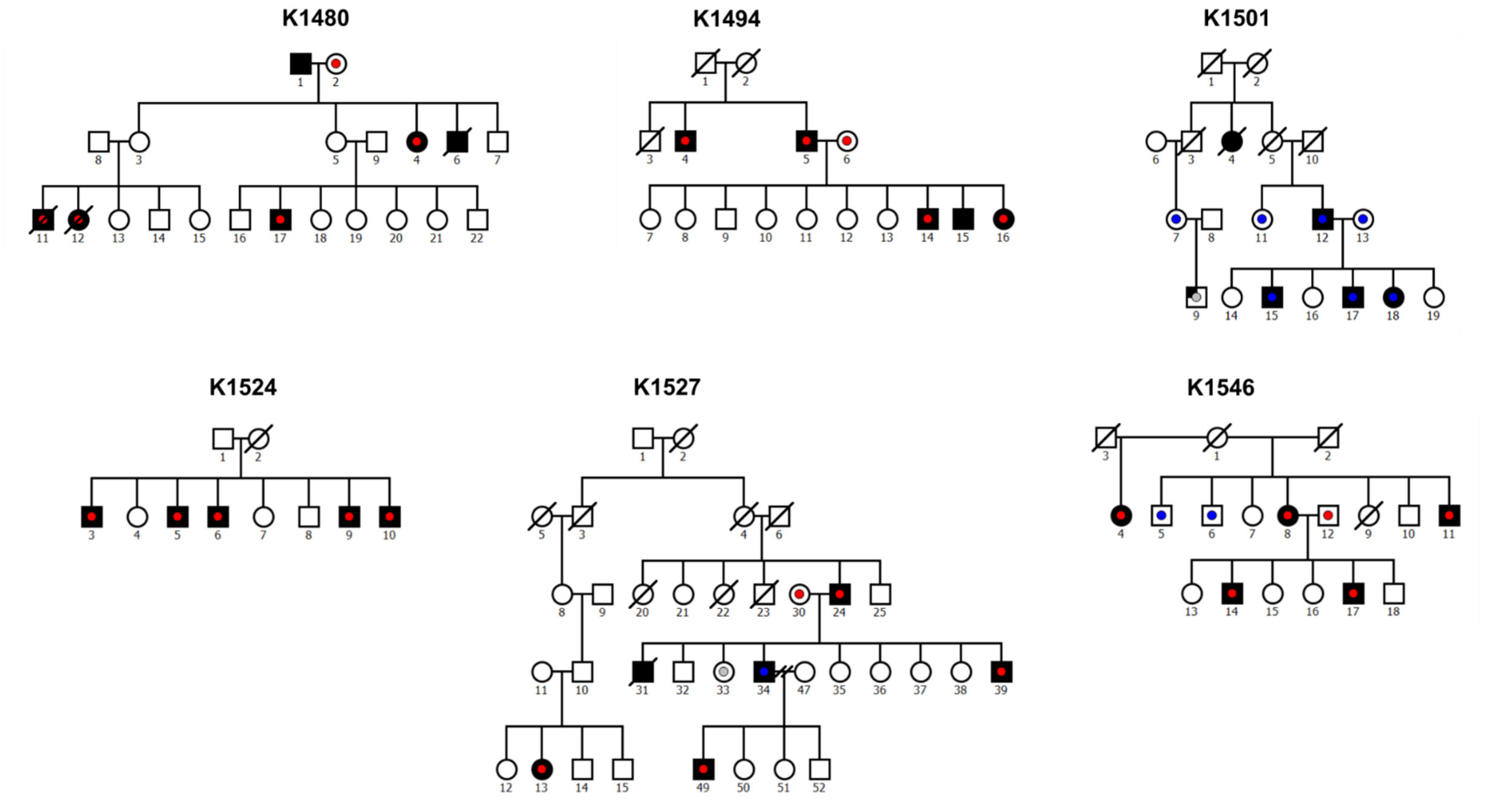

Supplementary Figure 2: A plot of the first two principal components of the 35 WGS samples and a background population from the 1000 Genome Project (2), re-generated from the output of *peddy* (3). AFR: African; AMR: admixed American; EAS: East Asian; EUR: European; SAS: South Asian.

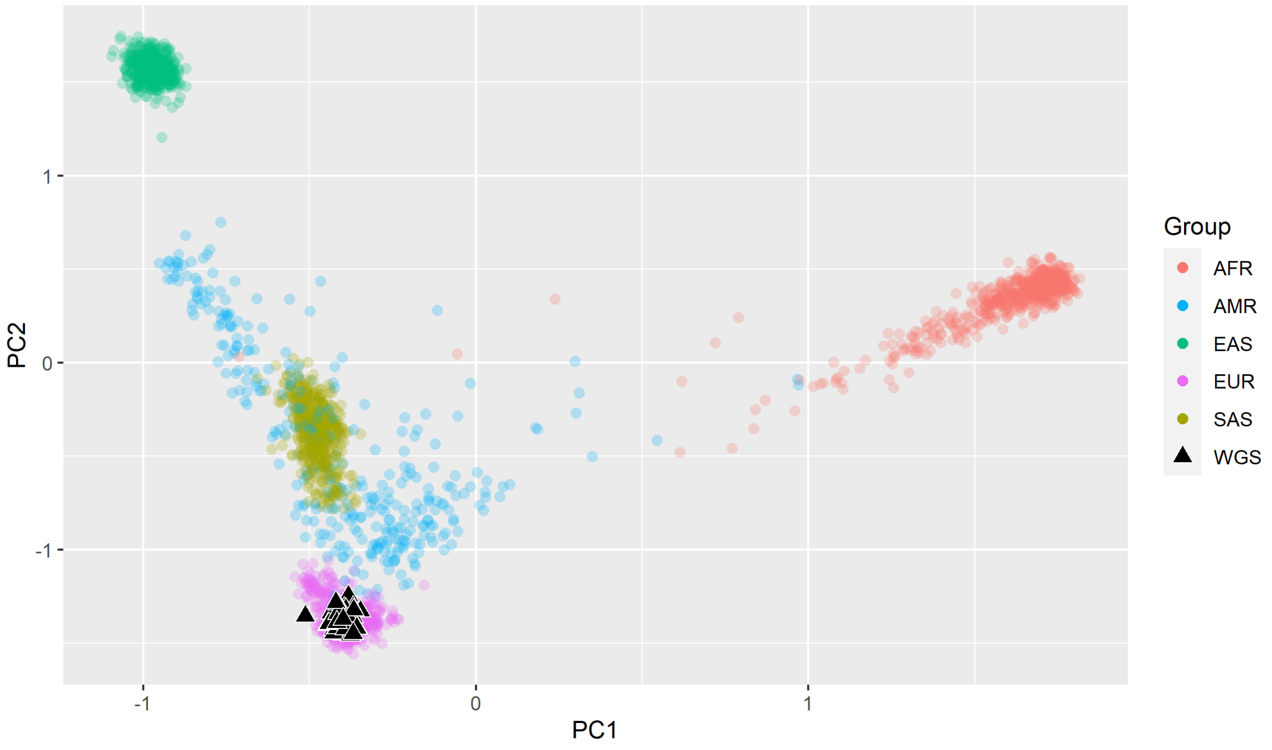

Supplementary Figure 3: A plot of the first two principal components from the XPAT (internal PCA) to identify technological stratification, with points highlighted for: (A) the batches; and (B) the pedigrees. Note: due to limitations on sample sizes, four additional pedigrees with WGS data from the same original cohort were included.

(A)

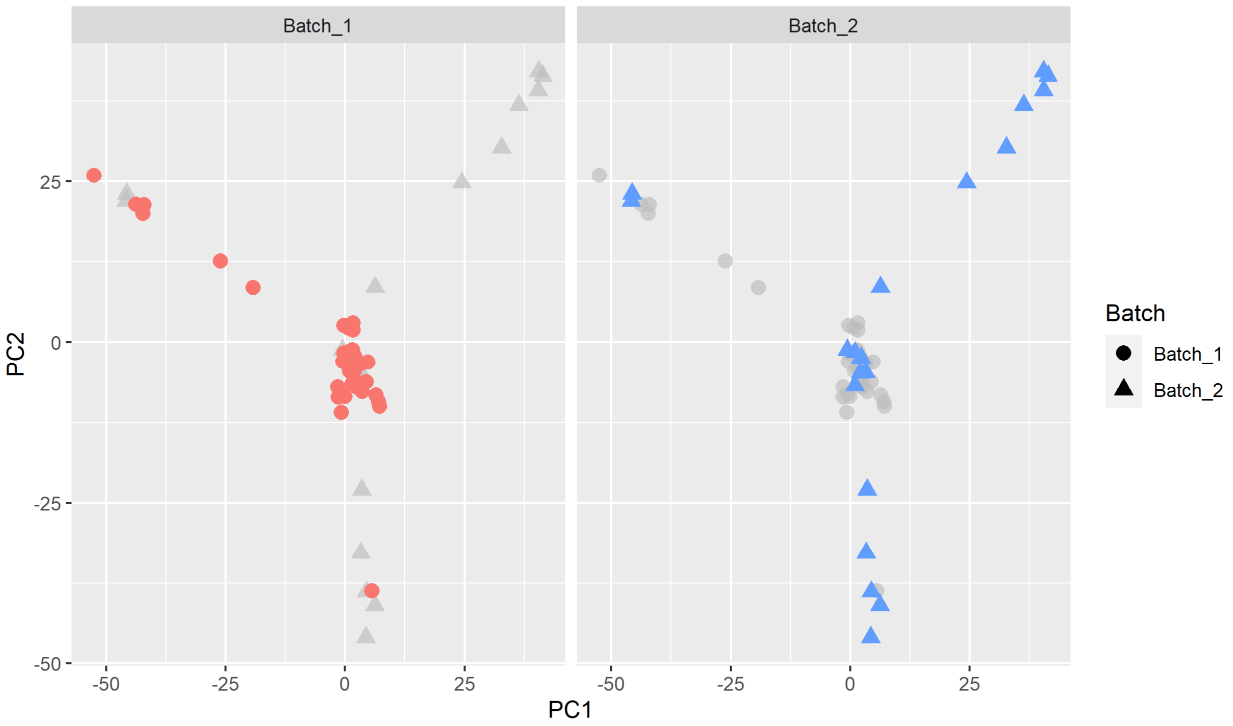

(B)

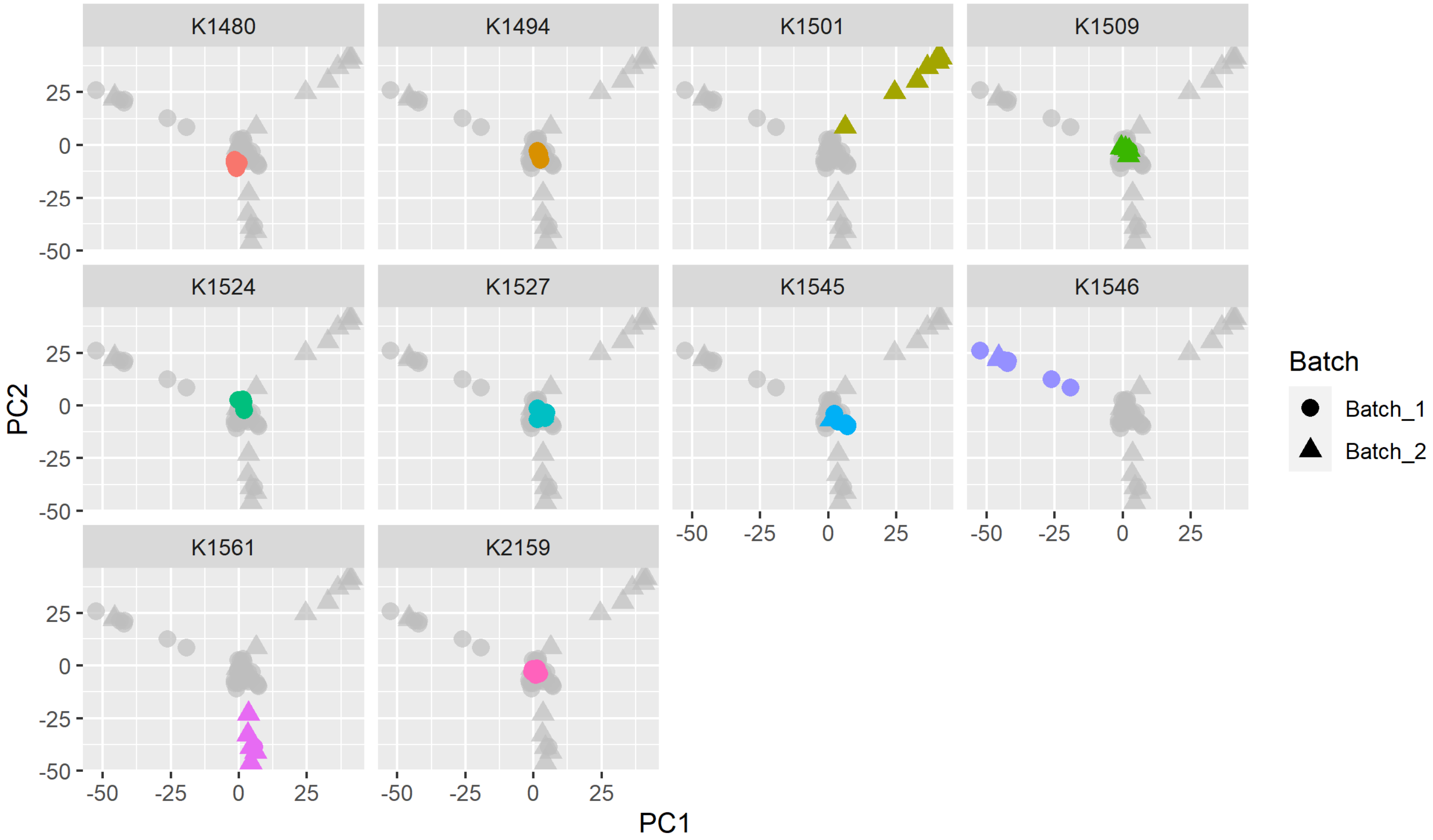

K1480
K1494
K1501
K1509
K1524
K1527
K1545
K1546
K1561
K2159
25
0
-25
-50
-50
-25
0
25
PC2
PC1
Batch
Batch_1
Batch_2

Supplementary Figure 4: A plot of pairs of the first ten principal components from the XPAT (internal PCA) to identify technological stratification, with points coloured by: (A) the batches; and (B) the pedigrees.

(A)

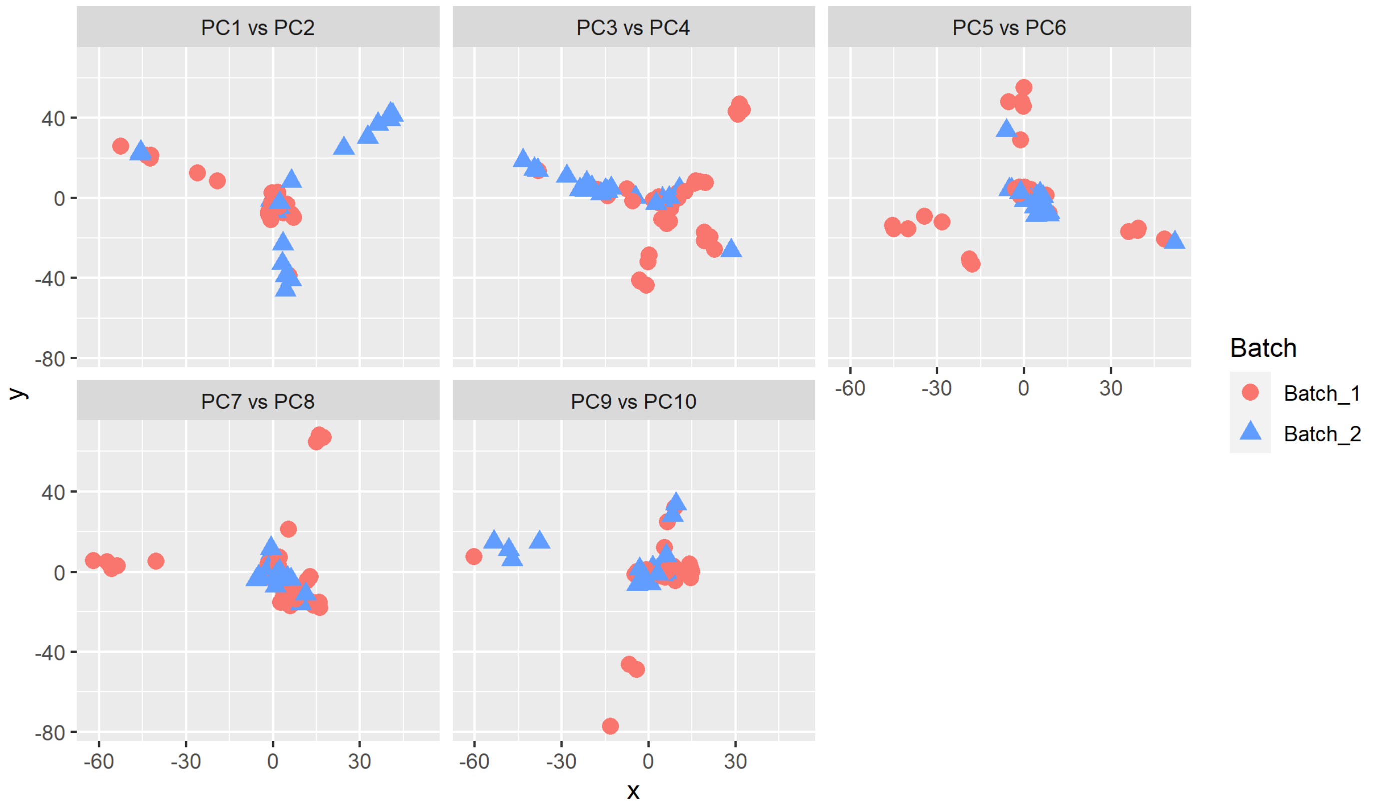

(B)

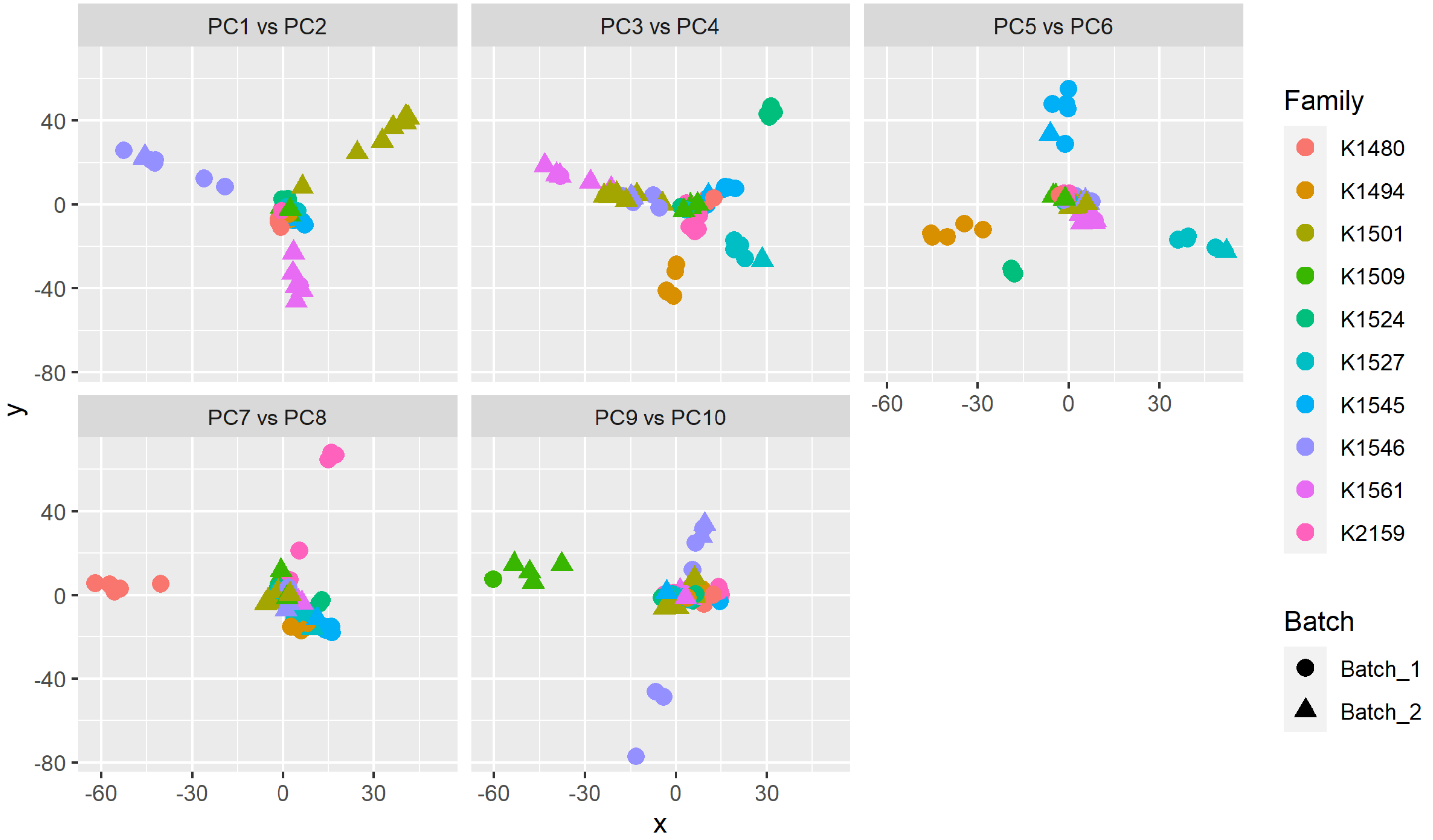

PC1 vs PC2
PC3 vs PC4
PC5 vs PC6
PC7 vs PC8
PC9 vs PC10
40
0
-40
-80
-60
-30
0
30
y
x
Family
K1480
K1494
K1501
K1509
K1524
K1527
K1545
K1546
K1561
K2159
Batch
Batch_1
Batch_2

Supplementary Figure 5: A workflow of the copy number variant calling pipeline applied to each sample. RLCR: repeat and low-complexity regions.

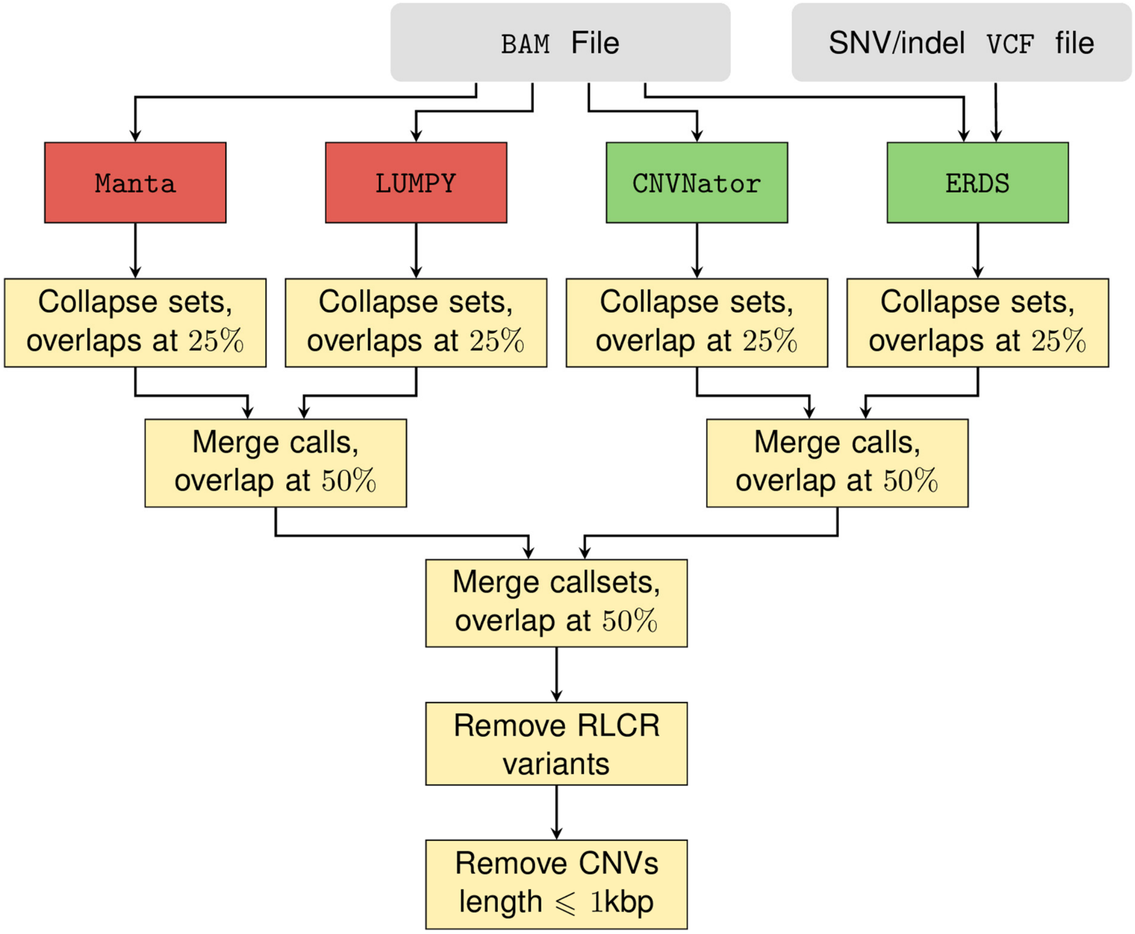

Supplementary References

1. Rees E, Kirov G (2021): Copy number variation and neuropsychiatric illness. *Current opinion in genetics & development*. 68:57-63.

2. Auton A, Brooks LD, Durbin RM, Garrison EP, Kang HM, Korbel JO, et al. (2015): A global reference for human genetic variation. *Nature*. 526:68-74.

3. Pedersen BS, Quinlan AR (2017): Who's Who? Detecting and Resolving Sample Anomalies in Human DNA Sequencing Studies with Peddy. *American journal of human genetics*. 100:406-413.
